# Supplementary material for: The social costs of tropical cyclones
Source: Nat Commun. 2023 Nov 23;14:7294. doi: 10.1038/s41467-023-43114-4 (PMC10667268; doi:10.1038/s41467-023-43114-4)
Supplement: Supplementary file 1 — Supplementary information [file 41467_2023_43114_MOESM1_ESM.pdf]

Supplementary Information (SI)
The Social Cost of Tropical Cyclones

Hazem Krichene[a], Thomas Vogt[a], Franziska Piontek[a], Tobias Geiger[a,b], Christof Schötz[a], and Christian Otto[a,*]

[a] Potsdam Institute for Climate Impact Research, Potsdam, Germany

[b] Deutscher Wetterdienst (DWD), Climate and Environment Consultancy, Potsdam, Germany

[*]Correspondence to: christian.otto@pik-potsdam.de

Supplementary Figures S1–S15

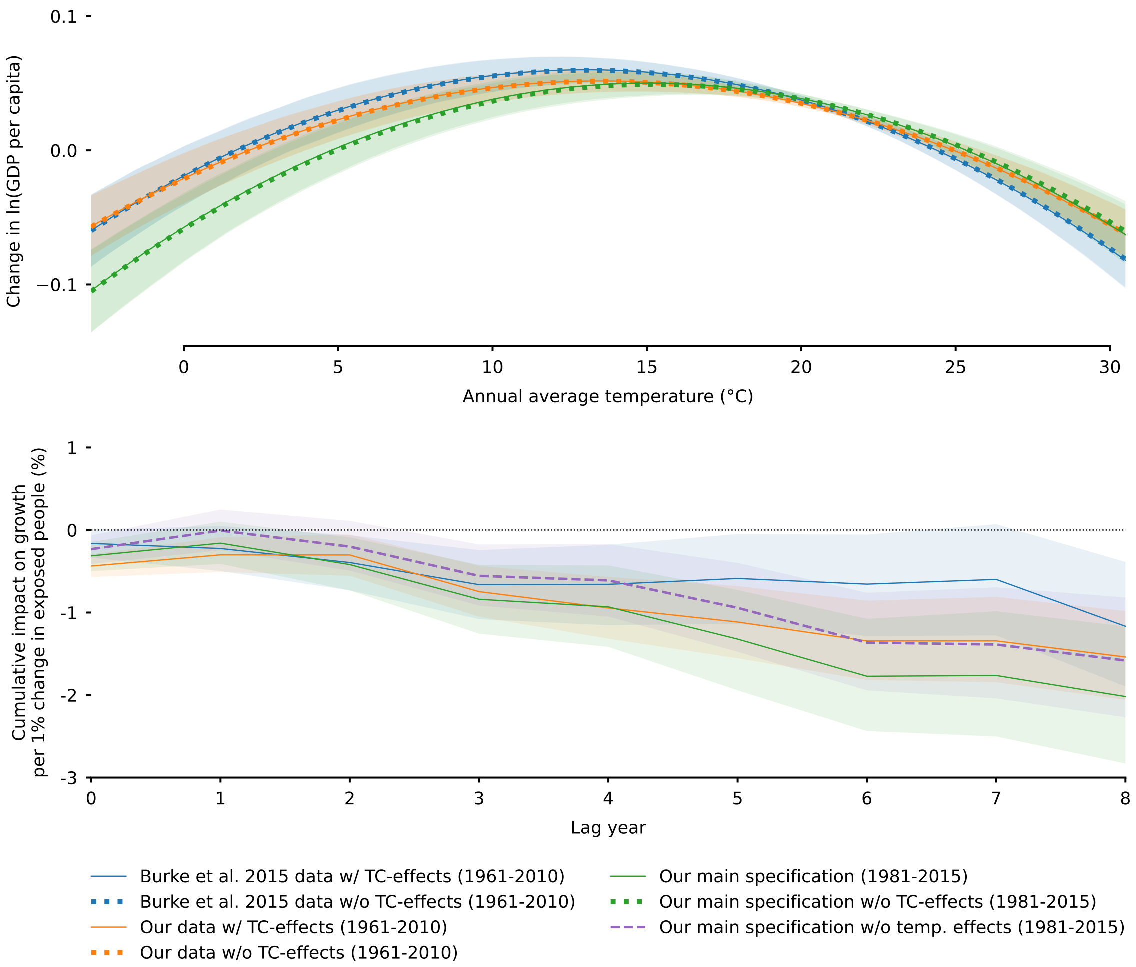

Fig. S1. Effects of annual average temperature and tropical cyclones (TCs) on economic production for different model configurations. Upper panel: Temperature effect on economic activity (as change in logarithm of per-capita GDP) found for the regression model of Eq. (1) with TC effect (solid lines) and without TC effects (dotted lines) for the periods 1961–2010 (orange) and 1981–2015 (green, main specification) as obtained from our data as well as obtained from the data used by Burke et al. in ref.[1] for the period 1961–2010. Shaded areas mark 90% confidence intervals from bootstrapping. **Lower panel:** Cumulative impact of TCs on economic activity per 1% change in exposed people as a function of the lag years for a maximum number of 8 lag years. Same colour and line codes as in upper panel. In addition the dashed line indicates the regression model without temperature effect.

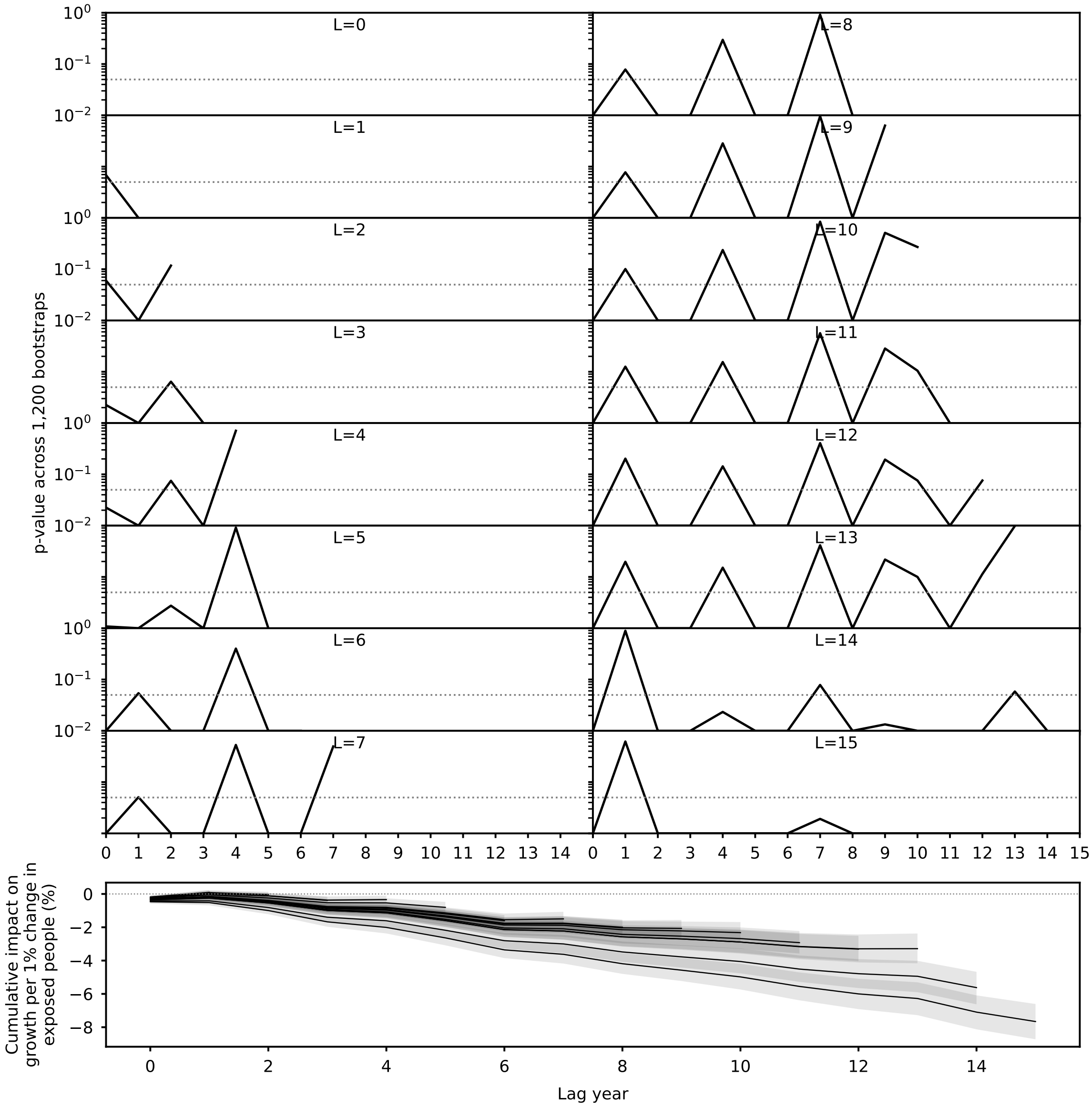

Fig. S2. Significance of TC-induced growth impacts for different lag numbers. The TC-induced growth impacts are not significant for lag numbers $7 \leq L \leq 13$ at a 5% level of significance, except for $L = 8$ and $L = 11$(top). This means that the cumulative effects remain almost unchanged for higher lag numbers while uncertainty increases (bottom). Based on this, we decided to use $L = 8$ lags as main specification.

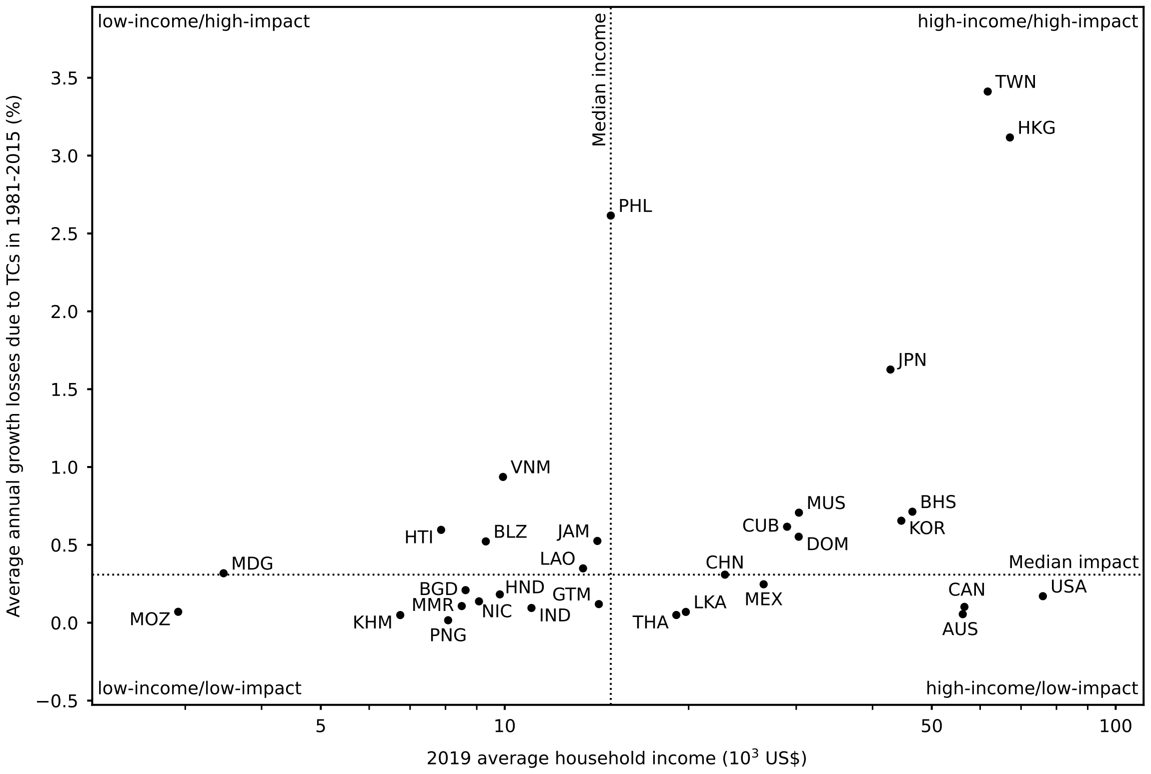

Fig. S3. Growth losses by tropical cyclones. Average annual growth losses due to tropical cyclones in 1981-2015; note log-scale on x-axis. The quadrants classify countries by above (high) and below (low) median income and losses across the exposed countries for which income data is available. The plot is for the main configuration with 8 lag years.

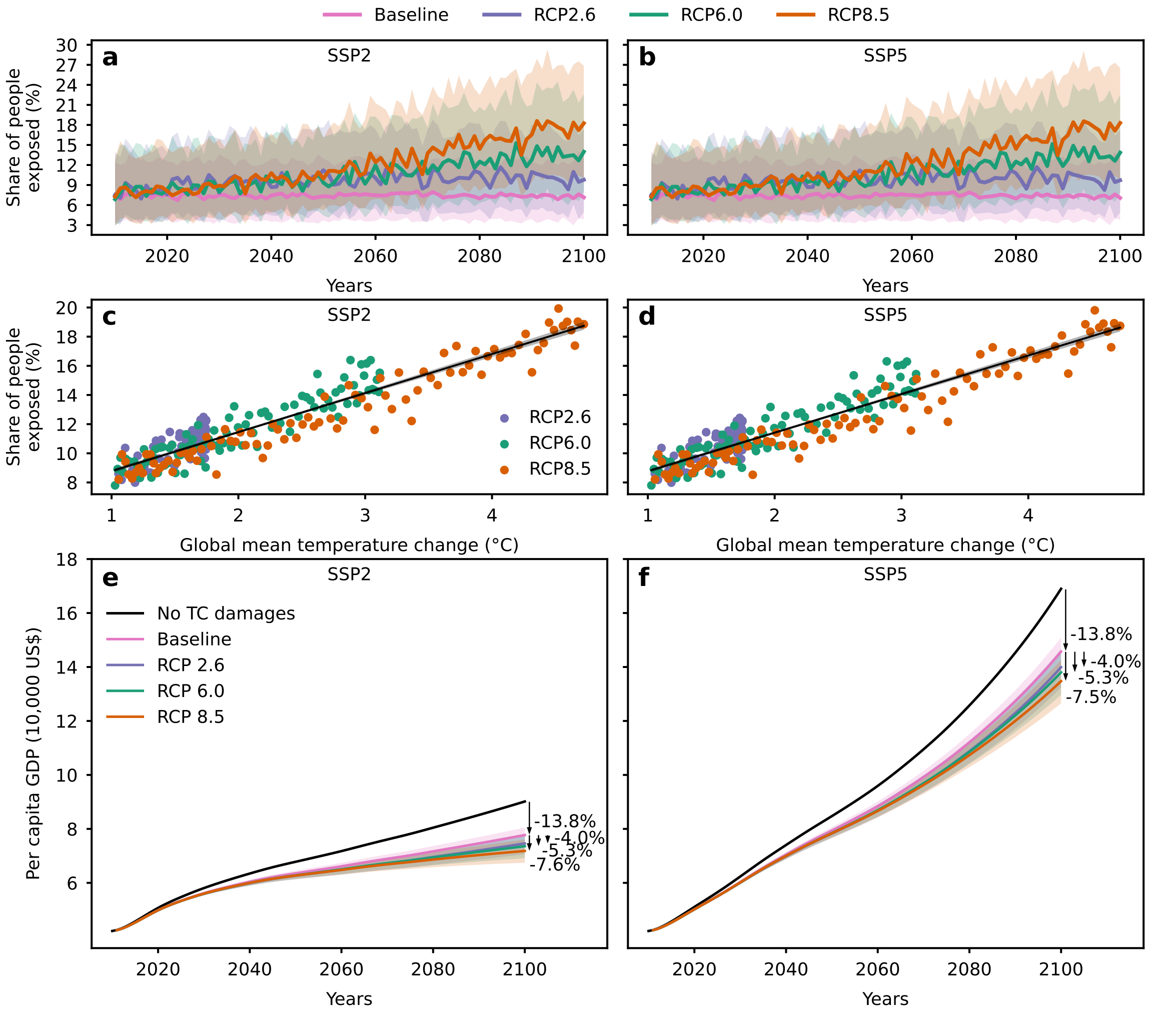

Fig. S4. Future population exposure and economic growth response to tropical cyclones impacts for the USA. Projected national shares of U.S. population exposed to tropical cyclones (TCs) and GDP projections accounting for TC impacts for the USA over the period 2010–2100 under a "no-further-climate-change" baseline climate, the Representative Concentration Pathways (RCPs) 2.6 (blue), 6.0 (orange), and 8.5 (red) as well as Shared Socioeconomic Pathways (SSPs) 2 (left column) and 5 (right column). **a, b,** Time series of the average shares of U.S. population exposed to strong winds from TCs. **c, d,** Average shares of U.S. population exposed to strong winds from TCs as a function of global mean temperature (GMT) change above pre-industrial levels. Markers denote annual shares of U.S. population averaged across 100 TC-realizations, 4 global circulation models. Black lines denote linear fits across RCPs and the shaded area indicates 90% confidence interval. **e, f,** Unperturbed GDP pathways as derived from the SSPs (black lines) and perturbed GDP pathways as obtained when additionally accounting for TC impacts on economic growth. Numbers indicate relative reductions in economic growth in percent. Shaded areas indicate 66% confidence intervals.

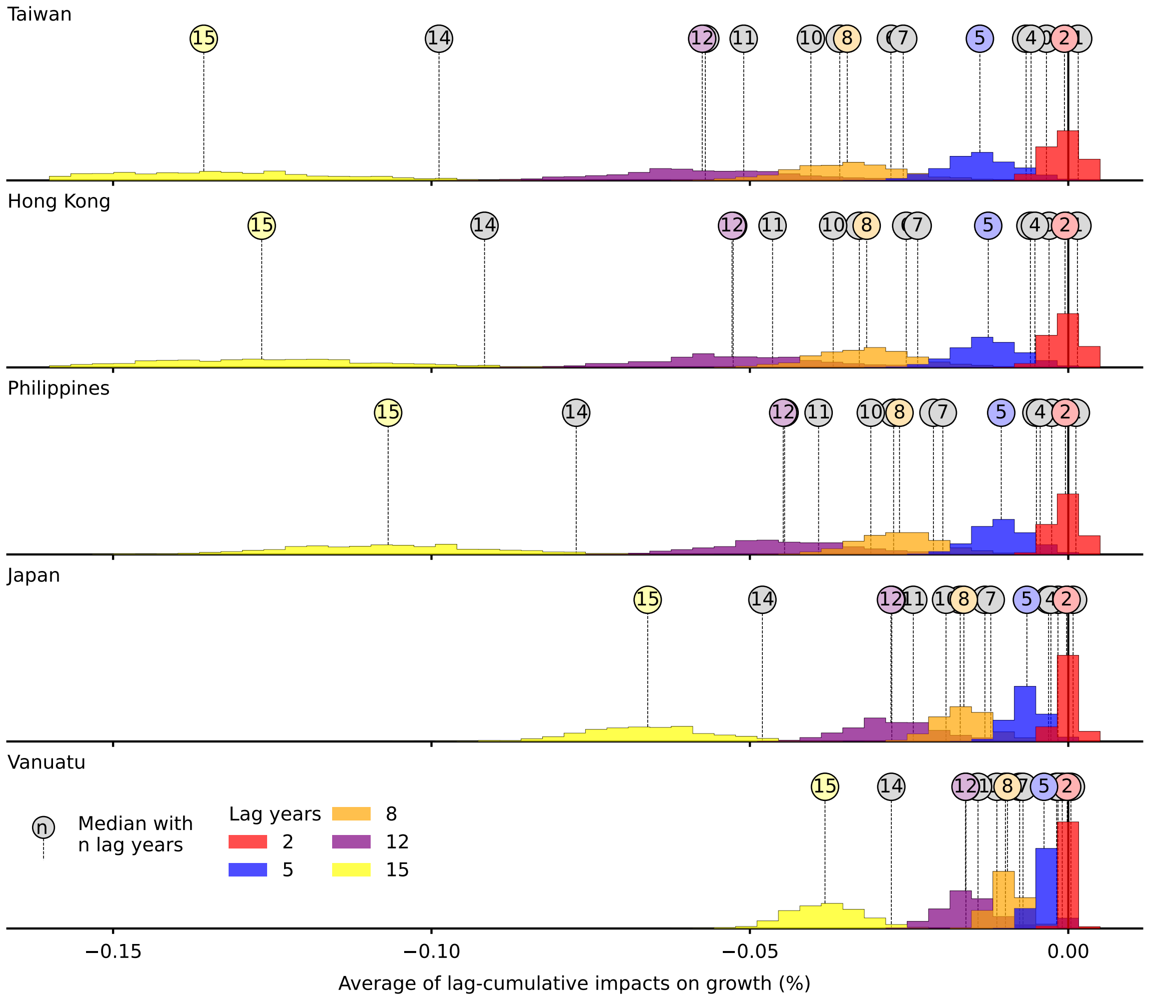

Fig. S5. Distributions of country-level growth responses to tropical cyclone strikes. Depicted are the distributions of country-level cumulative growth responses to tropical cyclones from 1,200 bootstraps for 2, 5, 8, 12, 15 lag-years (color code). Vertical lines with circles denote the median growth responses for 0–15 lag years. The order of countries follows the magnitude of the associated impacts from large impacts to small impacts (see x-axis scaling). Continued for other countries in Figs. S6 to S11.

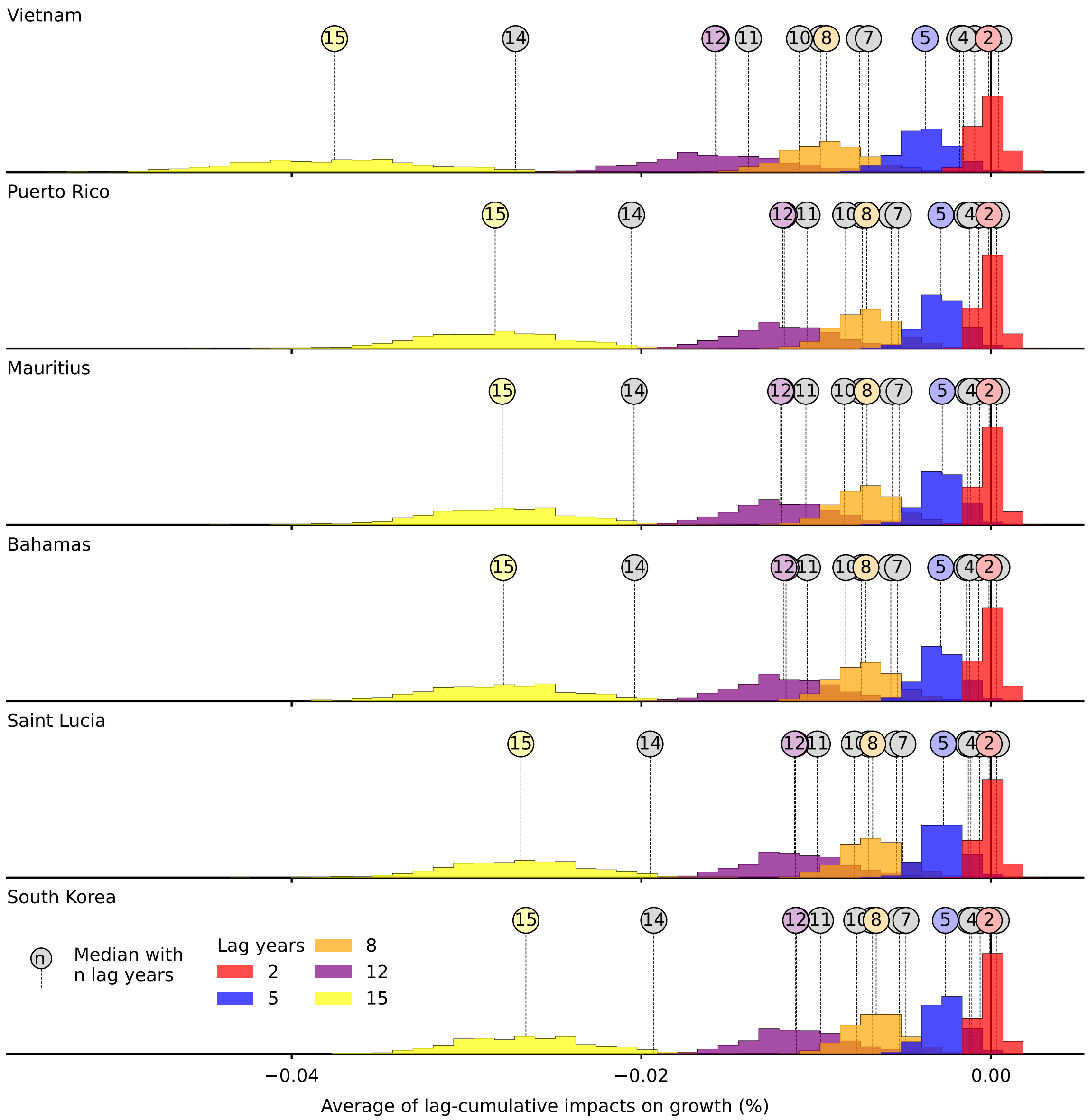

Fig. S6. Distributions of country-level growth responses to tropical cyclone strikes. Continuation of Fig. S5.

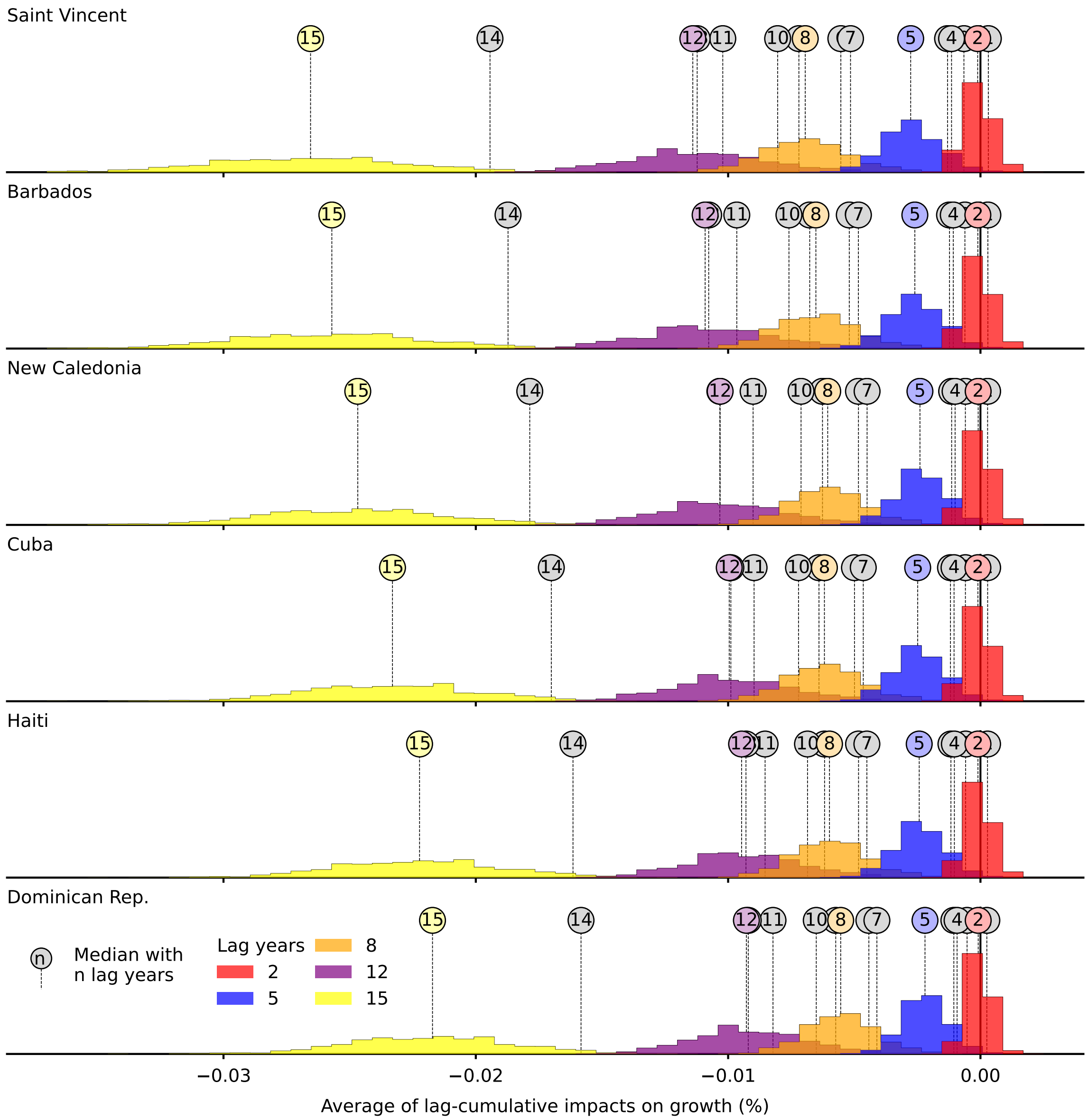

Fig. S7. Distributions of country-level growth responses to tropical cyclone strikes. Continuation of Fig. S5.

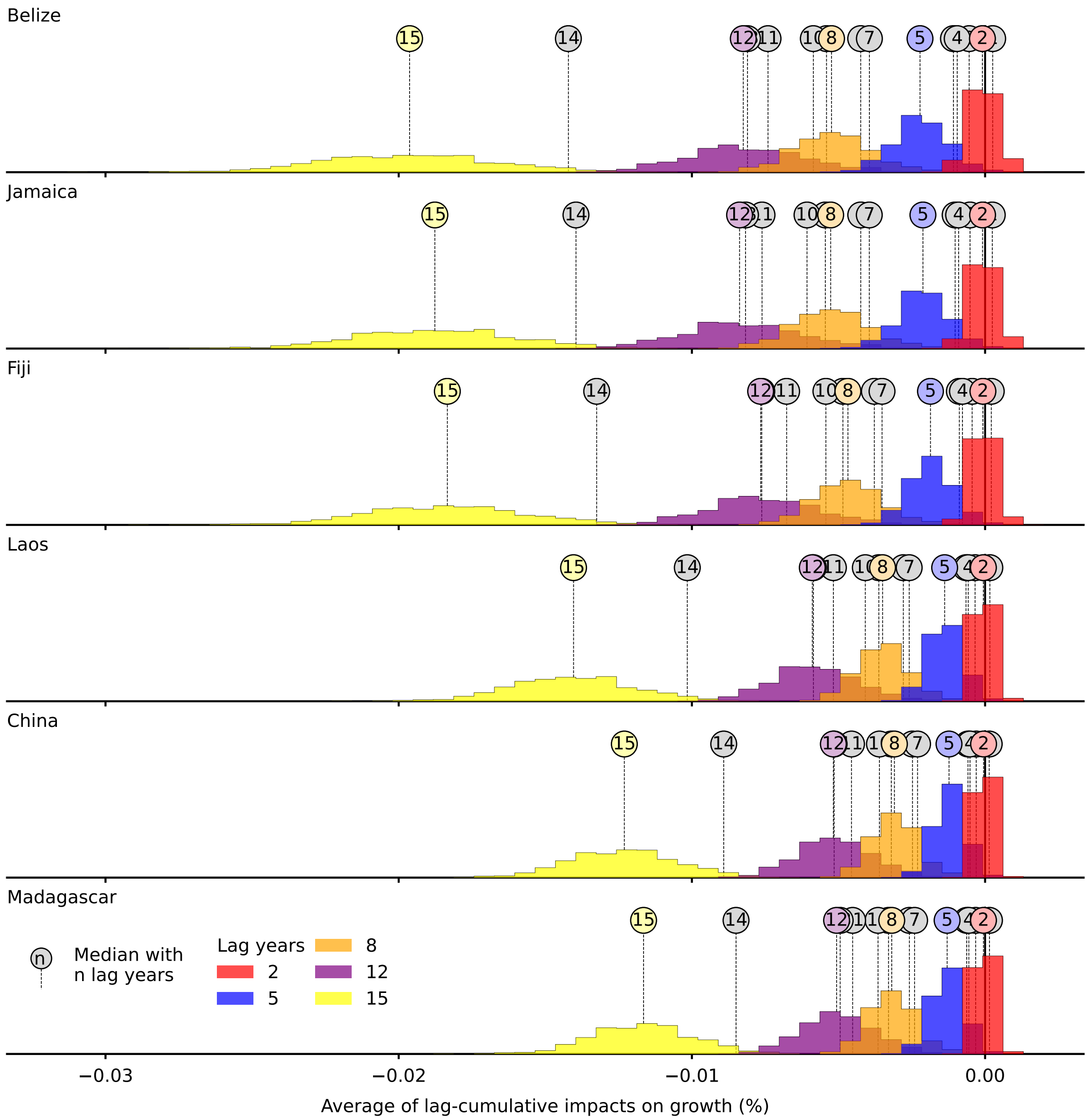

Fig. S8. Distributions of country-level growth responses to tropical cyclone strikes. Continuation of Fig. S5.

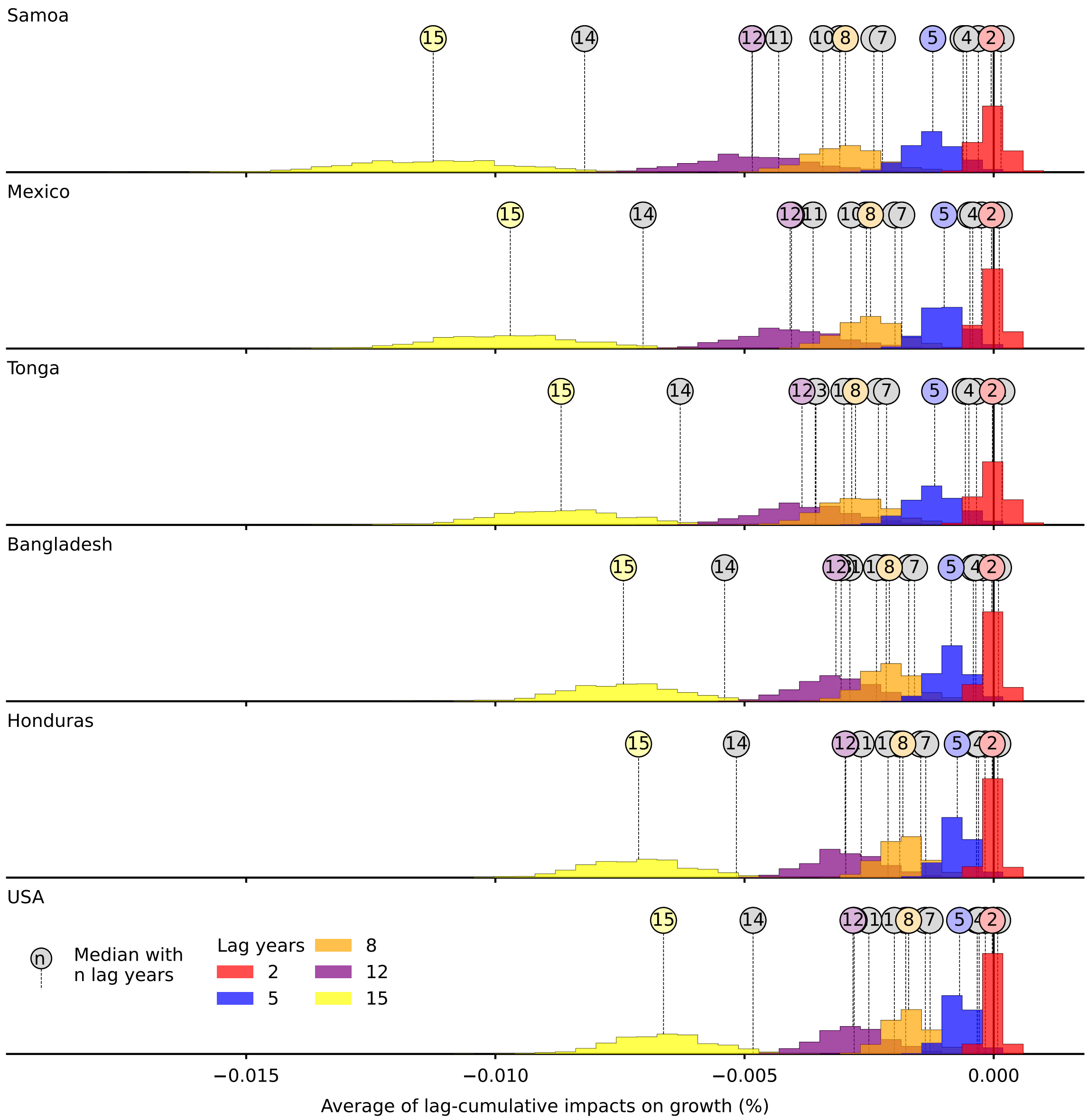

Fig. S9. Distributions of country-level growth responses to tropical cyclone strikes. Continuation of Fig. S5.

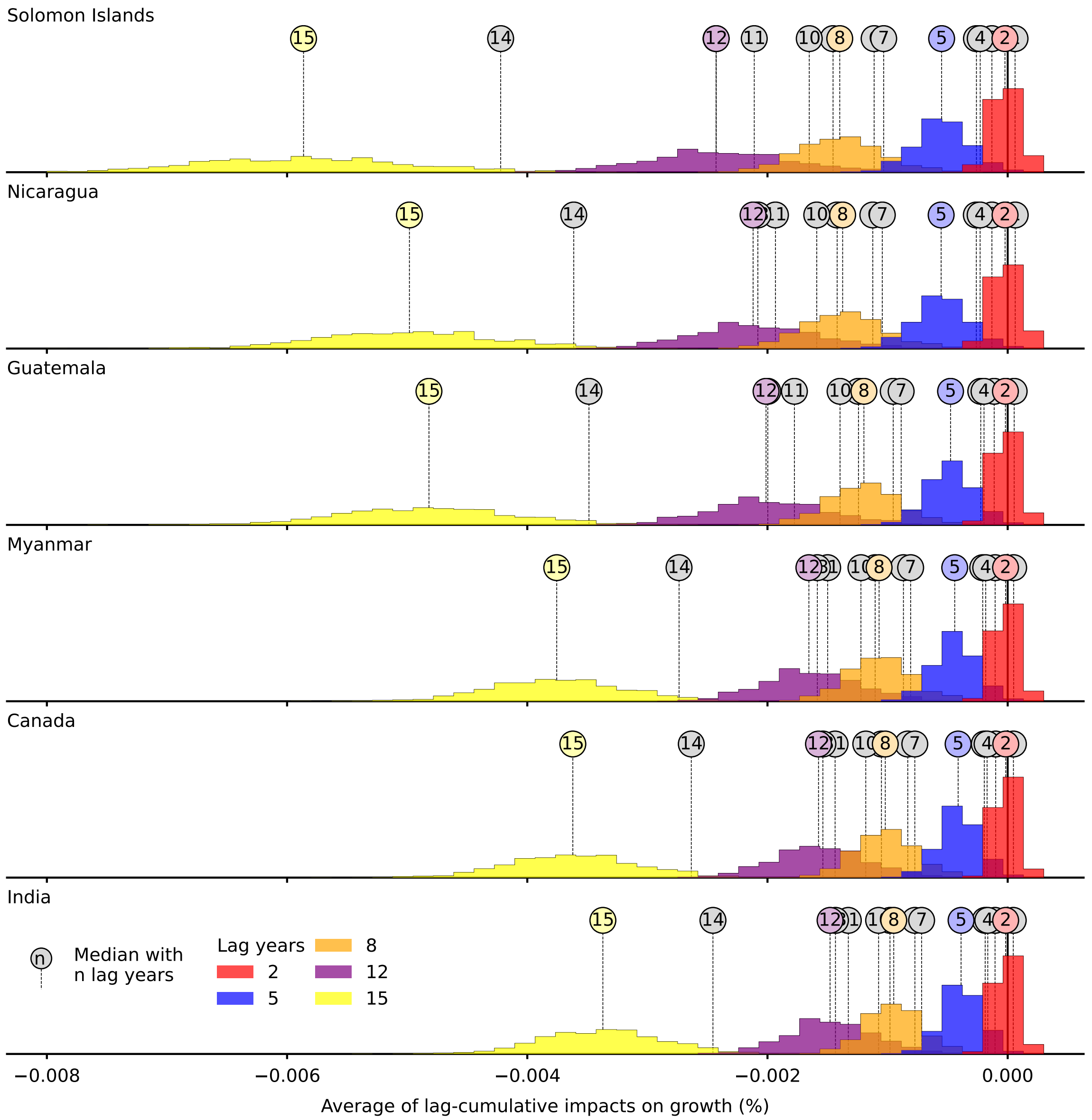

Fig. S10. Distributions of country-level growth responses to tropical cyclone strikes. Continuation of Fig. S5.

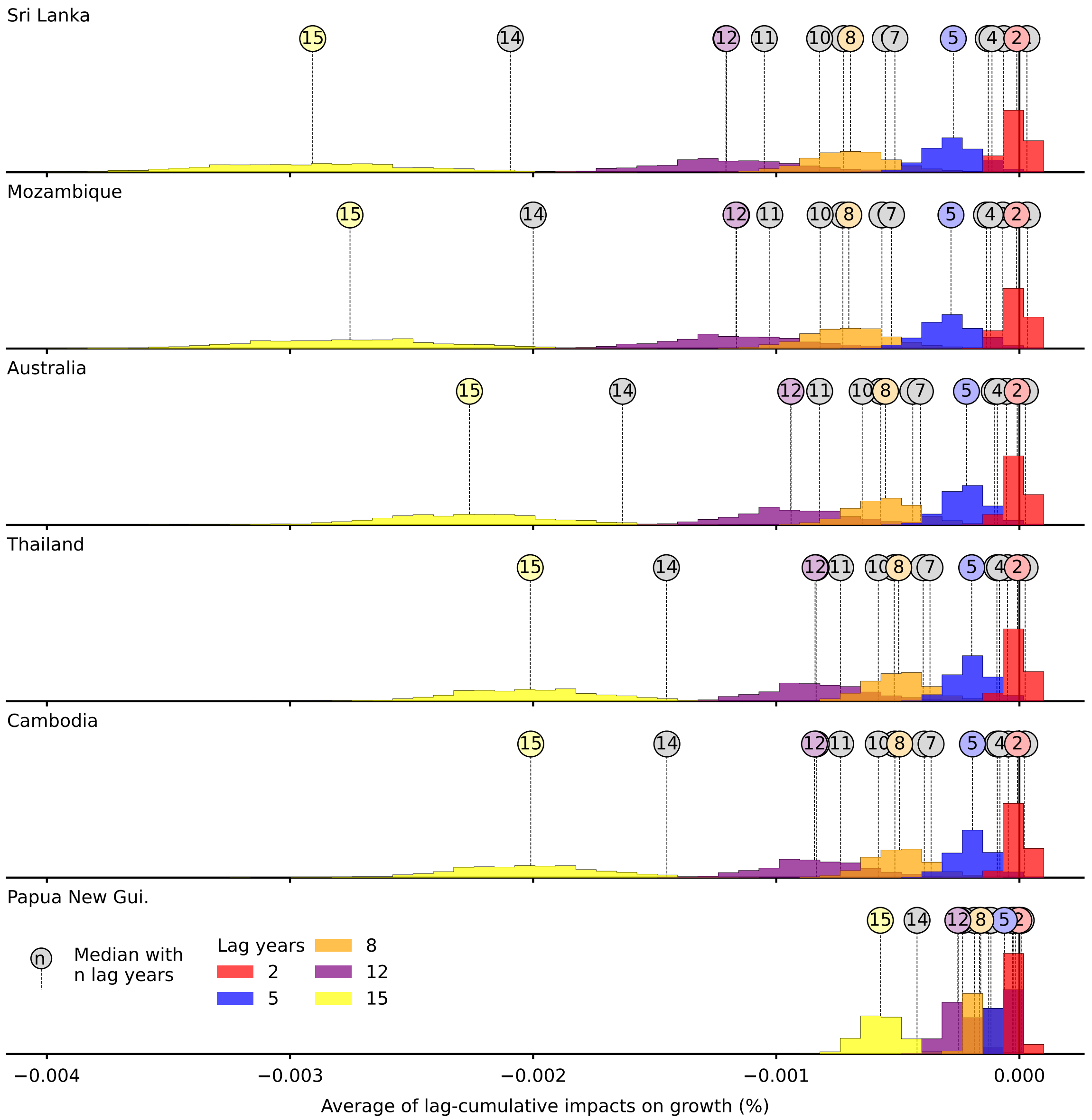

Fig. S11. Distributions of country-level growth responses to tropical cyclone strikes. Continuation of Fig. S5.

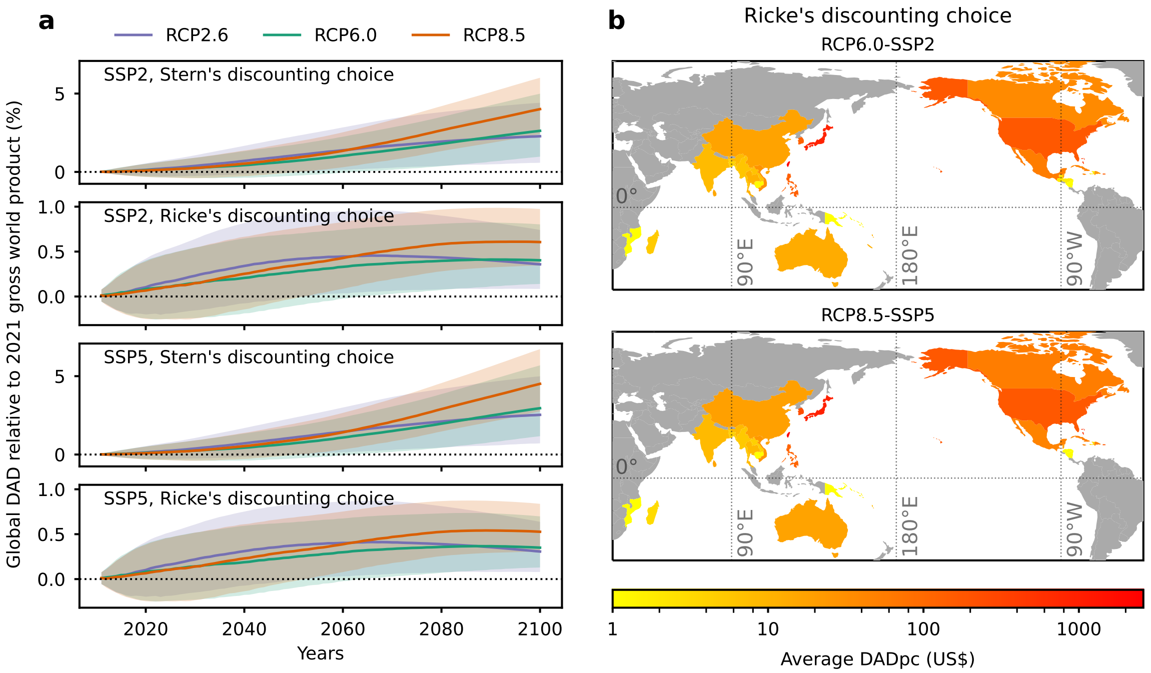

Fig. S12. Discounted annual damage (DAD) by tropical cyclones. a, Time series of median global DAD relative to 2021 global word product for Representative Concentration Pathway (RCP) 2.6 (blue lines), 6.0 (red lines), and 8.5 (orange lines) and for Shared Socioeconomic Pathways (SSP) 2 and 5, and Ricke's and Sterns choice of the growth adjusted discount-rate (Tbl. 1). Shaded areas depict 66% confidence intervals. **b,** Geographical distribution of median country-level DAD per-capita (DADpc) averaged over the period 2010–2100 for RCP6.0-SSP2 and RCP8.5-SSP5 and Ricke's discounting choice (see Tbl. 1).

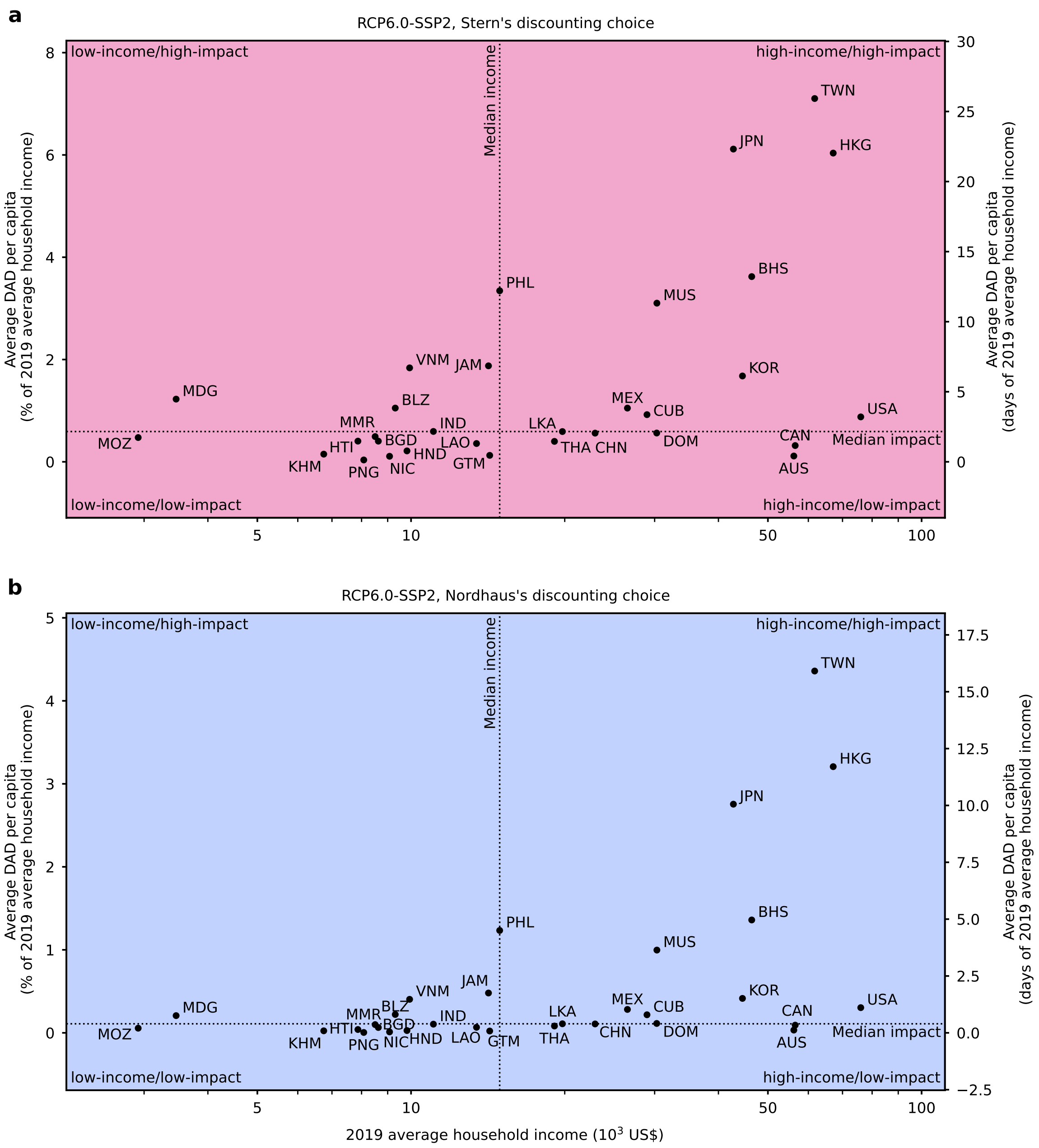

Fig. S13. Discounted annual damage (DAD) by tropical cyclones. Median average per-capita DAD relative to 2019 average household income (left y-axis); note log-scale on x-axis. The right y-axis shows median per-capita DAD in terms of days of average household income lost. The quadrants classify countries by above (high) and below (low) median income and per-capita DAD across the exposed countries for which income data is available. The plots are for SSP2 and RCP6.0 with Stern's (**a**) and Nordhaus's (**b**) discounting choice. See Fig. 2c for the main specification (Ricke's discounting choice).

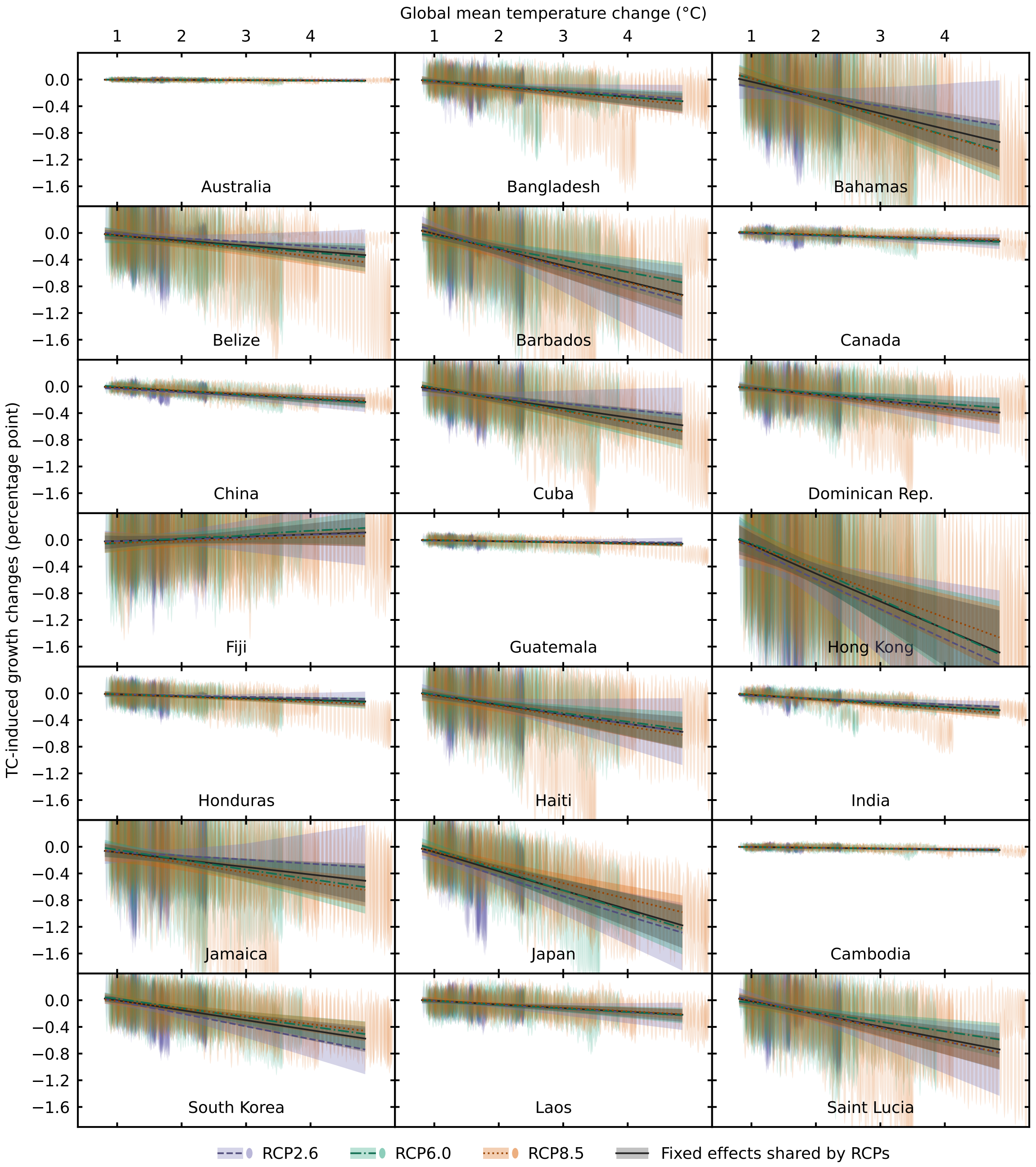

Fig. S14. Country-level temperature-dependent damage functions for tropical cyclone induced growth losses. The markers denote the 66% confidence range of annual relative growth losses across uncertainty dimensions 2, 4, and 5 (Tbl. 1) for three Representative Concentration Pathways (RCPs 2.6 (blue), 6.0 (orange) and 8.5 (red)). Black (colored) lines and shaded areas denote the fixed (random) effects in the mixed linear model and their 66% confidence intervals, respectively. Continued for other countries in Fig. S13. Parameters: 8 lags and Ricke's discounting choice (main specification).

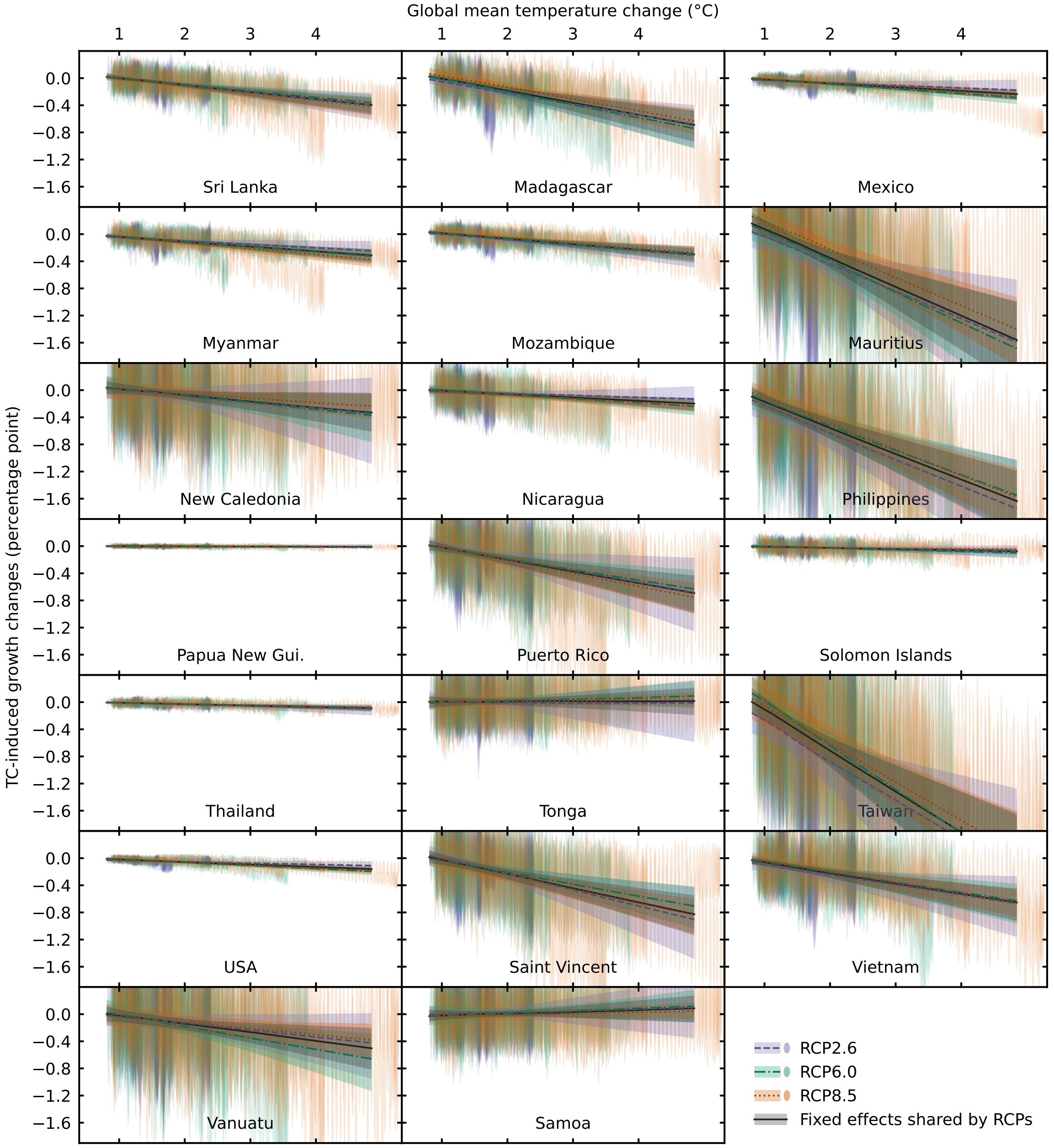

Fig. S15. Country-level temperature-dependent damage functions for tropical cyclone induced growth losses. Continuation of Fig. S14.

Supplementary Tables S1–S5

		Stern	Nordhaus	Ricke
SSP2	RCP2.6	1.25 (0.20–2.49)	0.47 (0.05–0.99)	0.36 (0.03–0.76)
		1.19% (0.19%–2.36%)	0.45% (0.05%–0.94%)	0.34% (0.03%–0.72%)
	RCP6.0	1.09 (0.16–2.22)	0.38 (0.01–0.84)	0.29 (-0.01–0.64)
		1.04% (0.15%–2.11%)	0.37% (0.01%–0.80%)	0.27% (-0.01%–0.61%)
	RCP8.5	1.54 (0.64–2.57)	0.52 (0.17–0.95)	0.38 (0.10–0.72)
		1.46% (0.61%–2.44%)	0.50% (0.16%–0.90%)	0.36% (0.10%–0.68%)
SSP5	RCP2.6	1.37 (0.24–2.74)	0.44 (0.04–0.93)	0.33 (0.02–0.70)
		1.27% (0.22%–2.55%)	0.41% (0.04%–0.86%)	0.31% (0.02%–0.65%)
	RCP6.0	1.21 (0.20–2.46)	0.36 (0.01–0.79)	0.26 (-0.01–0.60)
		1.12% (0.19%–2.29%)	0.34% (0.01%–0.74%)	0.24% (-0.01%–0.55%)
	RCP8.5	1.71 (0.74–2.83)	0.49 (0.15–0.89)	0.35 (0.09–0.66)
		1.59% (0.68%–2.63%)	0.46% (0.14%–0.83%)	0.33% (0.08%–0.62%)

Tbl. S1. Median global discounted annual damage averaged over the period 2010–2100 and 66% confidence intervals (in parentheses) as absolute numbers in trillion US$ and relative to global world product for Representative Concentration Pathways (RCPs) 2.6, 6.0, 8.5, Shared Socioeconomic Pathways (SSPs) 2 and 5 and the three discounting choices (Tbl. 1). See Fig. 2a for a visualization.

Country	ISO code	Income	Median	17th percentile	83rd percentile
Australia	AUS	56199	13 (0.02%)	-37 (-0.07%)	70 (0.12%)
Bangladesh	BGD	8628	4 (0.04%)	-6 (-0.07%)	13 (0.15%)
Bahamas	BHS	46481	458 (0.99%)	-497 (-1.07%)	1446 (3.11%)
Belize	BLZ	9312	14 (0.15%)	-53 (-0.56%)	131 (1.41%)
Barbados	BRB	–	151 (–)	-157 (–)	710 (–)
Canada	CAN	56548	37 (0.07%)	-66 (-0.12%)	206 (0.36%)
China	CHN	22934	17 (0.07%)	-1 (-0.00%)	36 (0.16%)
Cuba	CUB	28982	44 (0.15%)	-27 (-0.09%)	120 (0.42%)
Dominican Rep.	DOM	30282	23 (0.08%)	-50 (-0.17%)	120 (0.40%)
Fiji	FJI	–	-10 (–)	-124 (–)	94 (–)
Guatemala	GTM	14258	2 (0.01%)	-2 (-0.02%)	13 (0.09%)
Hong Kong	HKG	67123	1605 (2.39%)	-1302 (-1.94%)	4708 (7.01%)
Honduras	HND	9818	2 (0.02%)	-10 (-0.10%)	22 (0.23%)
Haiti	HTI	7869	2 (0.02%)	-6 (-0.08%)	14 (0.18%)
India	IND	11061	8 (0.07%)	0 (0.00%)	17 (0.15%)
Jamaica	JAM	14179	47 (0.33%)	-118 (-0.83%)	209 (1.47%)
Japan	JPN	42791	882 (2.06%)	200 (0.47%)	1800 (4.21%)
Cambodia	KHM	6746	1 (0.02%)	-1 (-0.02%)	4 (0.06%)
South Korea	KOR	44577	124 (0.28%)	-186 (-0.42%)	523 (1.17%)
Laos	LAO	13435	6 (0.05%)	-7 (-0.05%)	20 (0.15%)
Saint Lucia	LCA	–	50 (–)	-53 (–)	218 (–)
Sri Lanka	LKA	19790	15 (0.08%)	-12 (-0.06%)	39 (0.20%)
Madagascar	MDG	3466	5 (0.15%)	-1 (-0.04%)	13 (0.37%)
Mexico	MEX	26534	55 (0.21%)	6 (0.02%)	111 (0.42%)
Myanmar	MMR	8505	6 (0.07%)	-1 (-0.01%)	13 (0.15%)
Mozambique	MOZ	2921	1 (0.04%)	-2 (-0.06%)	4 (0.14%)
Mauritius	MUS	30307	216 (0.71%)	-314 (-1.04%)	692 (2.28%)
New Caledonia	NCL	–	12 (–)	-568 (–)	486 (–)
Nicaragua	NIC	9076	1 (0.01%)	-13 (-0.14%)	18 (0.20%)
Philippines	PHL	14914	139 (0.93%)	-2 (-0.02%)	299 (2.01%)
Papua New Gui.	PNG	8080	0 (0.00%)	-2 (-0.02%)	2 (0.03%)
Puerto Rico	PRI	–	141 (–)	-199 (–)	569 (–)
Solomon Islands	SLB	–	1 (–)	-6 (–)	10 (–)
Thailand	THA	19093	11 (0.06%)	-4 (-0.02%)	28 (0.15%)
Tonga	TON	–	-11 (–)	-82 (–)	50 (–)
Taiwan	TWN	61739	2046 (3.31%)	-558 (-0.90%)	4923 (7.97%)
USA	USA	76003	170 (0.22%)	49 (0.06%)	325 (0.43%)
Saint Vincent	VCT	–	61 (–)	-80 (–)	283 (–)
Vietnam	VNM	9935	29 (0.29%)	-11 (-0.11%)	67 (0.68%)
Vanuatu	VUT	–	11 (–)	-119 (–)	128 (–)
Samoa	WSM	–	-5 (–)	-86 (–)	64 (–)

Tbl. S2. Median country-level per-capita discounted annual damage (DAD) and 66% (17th – 83rd percentile) confidence intervals as obtained when averaging over the period 2010–2100 as absolute values in US$ and relative to 2019 average household income (also in US$) for the main specification (RCP6.0-SSP2 and Ricke's discounting choice). Country names are also given as 3-letter codes according to ISO 3166-1. See Fig. 2b and Fig. 2c for a visualization.

Country	Intercept			Slope		
	Mean	17th	83rd	Mean	17th	83rd
Australia	0.001	-0.007	0.010	-0.004	-0.008	0.001
Bahamas	0.207	0.021	0.382	-0.242	-0.347	-0.138
Bangladesh	0.052	-0.013	0.113	-0.080	-0.118	-0.042
Barbados	0.243	0.084	0.408	-0.248	-0.348	-0.151
Belize	0.046	-0.057	0.143	-0.082	-0.134	-0.033
Cambodia	0.008	-0.004	0.020	-0.012	-0.019	-0.004
Canada	0.035	0.012	0.061	-0.034	-0.049	-0.017
China	0.039	0.015	0.063	-0.057	-0.076	-0.038
Cuba	0.099	0.015	0.182	-0.143	-0.199	-0.087
Dominican Rep.	0.071	0.004	0.144	-0.097	-0.139	-0.054
Fiji	-0.069	-0.212	0.076	0.039	-0.024	0.108
Guatemala	0.005	-0.015	0.025	-0.013	-0.023	-0.004
Haiti	0.114	0.023	0.224	-0.145	-0.214	-0.078
Honduras	0.013	-0.031	0.059	-0.030	-0.051	-0.010
Hong Kong	0.351	-0.046	0.716	-0.434	-0.641	-0.220
India	0.033	0.009	0.056	-0.060	-0.079	-0.041
Jamaica	0.037	-0.101	0.198	-0.119	-0.204	-0.037
Japan	0.210	0.094	0.322	-0.289	-0.375	-0.204
Laos	0.043	-0.005	0.095	-0.056	-0.084	-0.029
Madagascar	0.171	0.093	0.245	-0.181	-0.242	-0.118
Mauritius	0.517	0.227	0.797	-0.441	-0.617	-0.259
Mexico	0.037	0.007	0.066	-0.057	-0.079	-0.036
Mozambique	0.097	0.054	0.137	-0.083	-0.110	-0.055
Myanmar	0.032	-0.004	0.067	-0.073	-0.099	-0.047
New Caledonia	0.114	-0.047	0.270	-0.095	-0.180	-0.004
Nicaragua	0.039	-0.023	0.104	-0.051	-0.083	-0.020
Papua New Gui.	0.000	-0.008	0.008	-0.002	-0.006	0.001
Philippines	0.211	0.023	0.404	-0.388	-0.517	-0.259
Puerto Rico	0.156	0.039	0.287	-0.179	-0.259	-0.099
Saint Lucia	0.179	0.045	0.309	-0.195	-0.277	-0.115
Saint Vincent	0.190	0.048	0.332	-0.213	-0.300	-0.126
Samoa	-0.039	-0.159	0.099	0.025	-0.045	0.087
Solomon Islands	0.010	-0.014	0.033	-0.018	-0.032	-0.005
South Korea	0.152	0.061	0.237	-0.152	-0.207	-0.097
Sri Lanka	0.108	0.051	0.168	-0.106	-0.143	-0.069
Taiwan	0.509	0.185	0.869	-0.621	-0.855	-0.376
Thailand	0.013	-0.004	0.033	-0.022	-0.034	-0.010
Tonga	0.001	-0.127	0.124	0.003	-0.061	0.063
USA	0.017	-0.000	0.033	-0.037	-0.049	-0.025
Vanuatu	0.102	-0.057	0.264	-0.125	-0.216	-0.040
Vietnam	0.097	0.009	0.196	-0.162	-0.229	-0.096

Tbl. S3. Intercepts and slopes together with their 66% (17th – 83rd percentile) confidence intervals of the country-specific temperature-dependent damage functions as obtained as the fixed effects shared by Representative Concentration Pathways (RCPs) 2.6, 6.0, and 8.5 in a mixed-effects linear model fitted across uncertainty dimensions 2, 4 and 5 (Tbl. 1). Intercepts and slopes are given in percentage points and percentage points per 1 °C of global mean temperature change, respectively. Parameters: 8 lag years and Ricke's discounting choice (Tbl. 1) (main specification).

	RCP		Global	USA	Japan	China	Taiwan	India
Stern	2.6	w/ TCs	1555 (927 – 2390) [+16%]	147 (59 – 291) [+37%]	40 (25 – 59) [+327%]	125 (65 – 207) [+40%]	40 (28 – 55) [+160%]	389 (248 – 585) [+8%]
		w/o TCs	1336 (767 – 2128)	108 (27 – 244)	9 (3 – 17)	89 (38 – 161)	15 (11 – 21)	360 (226 – 551)
	6.0	w/ TCs	1654 (1122 – 2361) [+16%]	149 (72 – 242) [+41%]	43 (27 – 67) [+452%]	112 (60 – 189) [+53%]	40 (28 – 55) [+160%]	399 (293 – 580) [+7%]
		w/o TCs	1428 (940 – 2078)	106 (38 – 192)	8 (2 – 15)	73 (31 – 137)	15 (11 – 21)	372 (269 – 545)
	8.5	w/ TCs	2284 (1557 – 3228) [+12%]	318 (185 – 433) [+16%]	62 (40 – 88) [+205%]	208 (110 – 323) [+25%]	43 (31 – 60) [+135%]	448 (324 – 662) [+6%]
		w/o TCs	2048 (1363 – 2945)	275 (145 – 384)	20 (9 – 38)	167 (75 – 273)	18 (13 – 25)	422 (300 – 627)
Nordhaus	2.6	w/ TCs	314 (185 – 509) [+21%]	33 (12 – 69) [+41%]	10 (7 – 16) [+394%]	24 (13 – 41) [+46%]	11 (7 – 17) [+191%]	72 (45 – 113) [+10%]
		w/o TCs	260 (146 – 440)	24 (5 – 57)	2 (0 – 4)	17 (7 – 31)	4 (3 – 5)	66 (40 – 104)
	6.0	w/ TCs	321 (208 – 484) [+21%]	31 (14 – 53) [+50%]	11 (7 – 17) [+596%]	20 (10 – 34) [+65%]	11 (8 – 18) [+213%]	70 (50 – 107) [+9%]
		w/o TCs	264 (166 – 407)	21 (5 – 41)	2 (0 – 3)	12 (4 – 24)	4 (3 – 5)	64 (45 – 97)
	8.5	w/ TCs	546 (337 – 886) [+16%]	72 (39 – 106) [+21%]	16 (10 – 25) [+283%]	38 (19 – 64) [+33%]	16 (10 – 29) [+228%]	104 (65 – 182) [+10%]
		w/o TCs	471 (284 – 771)	59 (29 – 90)	4 (2 – 8)	29 (12 – 50)	5 (3 – 8)	94 (59 – 163)
Ricke	2.6	w/ TCs	212 (127 – 341) [+21%]	22 (8 – 46) [+42%]	8 (5 – 11) [+410%]	17 (9 – 28) [+47%]	7 (5 – 11) [+191%]	49 (31 – 75) [+9%]
		w/o TCs	175 (100 – 294)	16 (3 – 38)	1 (0 – 3)	12 (5 – 21)	3 (2 – 4)	45 (28 – 69)
	6.0	w/ TCs	212 (138 – 318) [+22%]	20 (8 – 35) [+53%]	8 (5 – 12) [+634%]	14 (7 – 23) [+68%]	8 (5 – 12) [+215%]	47 (33 – 70) [+9%]
		w/o TCs	173 (108 – 266)	13 (3 – 26)	1 (-0 – 2)	8 (3 – 16)	2 (2 – 3)	43 (30 – 64)
	8.5	w/ TCs	361 (222 – 588) [+17%]	46 (25 – 69) [+22%]	11 (7 – 17) [+294%]	25 (13 – 42) [+35%]	11 (7 – 20) [+234%]	69 (44 – 120) [+10%]
		w/o TCs	309 (186 – 509)	38 (18 – 58)	3 (1 – 5)	19 (8 – 33)	3 (2 – 5)	62 (39 – 107)

Tbl. S4. Median social cost of carbon with and without the effects of tropical cyclones in US$/tCO2 together with 66% confidence intervals (in parentheses) for Representative Concentration Pathways (RCPs) 2.6, 6.0, 8.5 as obtained for Shared Socioeconomic Pathway (SSP) 2 as well as Stern's, Nordhaus's and Ricke's discounting choices (Tbl. 1), globally and for the 5 countries with the highest absolute increase in SCC due to TCs according to our main specification. The relative increases of the median SCC values when including TC effects are given in brackets. See Tbl. S5 for the corresponding SSP 5 data.

	RCP		Global	USA	Japan	China	Taiwan	India
Stern	2.6	w/ TCs	1255 (724 – 2003) [+21%]	170 (69 – 330) [+36%]	48 (31 – 71) [+322%]	107 (56 – 175) [+41%]	35 (23 – 52) [+178%]	295 (182 – 457) [+8%]
		w/o TCs	1041 (570 – 1748)	125 (32 – 278)	11 (3 – 21)	76 (33 – 136)	13 (9 – 18)	273 (166 – 427)
	6.0	w/ TCs	1358 (898 – 1976) [+20%]	167 (82 – 277) [+40%]	52 (33 – 81) [+439%]	93 (51 – 157) [+54%]	38 (27 – 53) [+187%]	313 (227 – 454) [+7%]
		w/o TCs	1135 (717 – 1692)	119 (43 – 216)	10 (3 – 18)	61 (26 – 112)	13 (9 – 19)	291 (204 – 427)
	8.5	w/ TCs	2096 (1338 – 3039) [+15%]	435 (208 – 666) [+23%]	84 (48 – 122) [+247%]	193 (91 – 307) [+30%]	41 (30 – 57) [+135%]	352 (255 – 518) [+6%]
		w/o TCs	1818 (1142 – 2711)	354 (162 – 584)	24 (11 – 47)	149 (62 – 258)	17 (13 – 24)	331 (237 – 491)
Nordhaus	2.6	w/ TCs	212 (120 – 352) [+27%]	33 (12 – 67) [+41%]	10 (7 – 15) [+392%]	18 (10 – 30) [+47%]	9 (6 – 14) [+200%]	42 (27 – 67) [+9%]
		w/o TCs	167 (87 – 296)	24 (4 – 55)	2 (0 – 4)	13 (5 – 23)	3 (2 – 4)	39 (24 – 61)
	6.0	w/ TCs	214 (135 – 326) [+28%]	30 (13 – 51) [+50%]	10 (7 – 16) [+589%]	15 (8 – 25) [+67%]	10 (7 – 16) [+230%]	42 (30 – 63) [+9%]
		w/o TCs	167 (98 – 264)	20 (4 – 39)	2 (0 – 3)	9 (3 – 17)	3 (2 – 4)	39 (27 – 57)
	8.5	w/ TCs	395 (232 – 642) [+21%]	77 (36 – 127) [+27%]	17 (9 – 27) [+318%]	30 (14 – 49) [+38%]	14 (8 – 25) [+224%]	60 (39 – 103) [+10%]
		w/o TCs	328 (188 – 544)	61 (27 – 107)	4 (2 – 8)	21 (9 – 39)	4 (3 – 6)	55 (35 – 93)
Ricke	2.6	w/ TCs	144 (82 – 237) [+28%]	23 (8 – 45) [+42%]	7 (5 – 11) [+410%]	13 (7 – 21) [+48%]	6 (4 – 10) [+196%]	29 (19 – 44) [+9%]
		w/o TCs	112 (59 – 198)	16 (2 – 37)	1 (0 – 3)	9 (4 – 16)	2 (2 – 3)	26 (17 – 40)
	6.0	w/ TCs	141 (88 – 214) [+30%]	20 (8 – 34) [+54%]	7 (5 – 11) [+631%]	10 (5 – 17) [+71%]	7 (5 – 11) [+226%]	28 (20 – 41) [+9%]
		w/o TCs	108 (63 – 171)	13 (2 – 25)	1 (-0 – 2)	6 (2 – 11)	2 (1 – 3)	26 (18 – 38)
	8.5	w/ TCs	258 (152 – 418) [+21%]	49 (24 – 80) [+27%]	11 (6 – 18) [+321%]	19 (10 – 32) [+39%]	10 (6 – 17) [+228%]	40 (26 – 67) [+10%]
		w/o TCs	212 (122 – 352)	39 (17 – 67)	3 (1 – 5)	14 (6 – 25)	3 (2 – 4)	36 (23 – 60)

Tbl. S5. Median social cost of carbon with and without the effects of tropical cyclones in US$/tCO2 together with 66% confidence intervals (in parentheses) for Representative Concentration Pathways (RCPs) 2.6, 6.0, 8.5 as obtained for Shared Socioeconomic Pathway (SSP) 5 as well as Stern's, Nordhaus's and Ricke's discounting choices (Tbl. 1), globally and for the 5 countries with the highest absolute increase in SCC due to TCs according to our main specification. The relative increases of the median SCC values when including TC effects are given in brackets. See Tbl. S4 for the corresponding SSP 2 data.

Supplementary References

[SI1] Burke, M., Hsiang, S. M. & Miguel, E. Global non-linear effect of temperature on economic production. *Nature* **527**, 235–239 (2015). 10.1038/nature15725.
