# Supplementary material for: The social costs of tropical cyclones
Source: Nat Commun. 2023 Nov 23;14:7294. doi: 10.1038/s41467-023-43114-4 (PMC10667268; doi:10.1038/s41467-023-43114-4)
Supplement: Supplementary file 3 — Reporting Summary [file 41467_2023_43114_MOESM3_ESM.pdf]

nature portfolio

Corresponding author(s): Christian Otto

Last updated by author(s): Jun 21, 2023

Reporting Summary

Nature Portfolio wishes to improve the reproducibility of the work that we publish. This form provides structure for consistency and transparency in reporting. For further information on Nature Portfolio policies, see our Editorial Policies and the Editorial Policy Checklist.

Statistics

For all statistical analyses, confirm that the following items are present in the figure legend, table legend, main text, or Methods section.

n/a	Confirmed	
☐	☒	The exact sample size (n) for each experimental group/condition, given as a discrete number and unit of measurement
☐	☒	A statement on whether measurements were taken from distinct samples or whether the same sample was measured repeatedly
☐	☒	The statistical test(s) used AND whether they are one- or two-sided<br>*Only common tests should be described solely by name; describe more complex techniques in the Methods section.*
☒	☐	A description of all covariates tested
☐	☒	A description of any assumptions or corrections, such as tests of normality and adjustment for multiple comparisons
☐	☒	A full description of the statistical parameters including central tendency (e.g. means) or other basic estimates (e.g. regression coefficient) AND variation (e.g. standard deviation) or associated estimates of uncertainty (e.g. confidence intervals)
☐	☒	For null hypothesis testing, the test statistic (e.g. F, t, r) with confidence intervals, effect sizes, degrees of freedom and P value noted<br>*Give P values as exact values whenever suitable.*
☒	☐	For Bayesian analysis, information on the choice of priors and Markov chain Monte Carlo settings
☒	☐	For hierarchical and complex designs, identification of the appropriate level for tests and full reporting of outcomes
☒	☐	Estimates of effect sizes (e.g. Cohen's d, Pearson's r), indicating how they were calculated

Our web collection on statistics for biologists contains articles on many of the points above.

Software and code

Policy information about availability of computer code

Data collection	no software was used to collect the data
Data analysis	he model to generate the probabilistic TC tracks from climate model outputs is intellectual property of WindRiskTech (info@windrisktech.com) and cannot be shared publicly. All remaining code that was used i) for the regression analyses, ii) the generation of the future tropical cyclone (TC) exposure indicators, and iii) the calculation of damage estimates, and iv) to analyze the data and produce the figures was implemented in Python 3.9 (https://www.python.org/) with CLIMADA 3.3.3 (https://zenodo.org/record/7691855) and statsmodels 0.13.5 (https://www.statsmodels.org/), and is openly available from https://dx.doi.org/10.5281/zenodo.8056520.

For manuscripts utilizing custom algorithms or software that are central to the research but not yet described in published literature, software must be made available to editors and reviewers. We strongly encourage code deposition in a community repository (e.g. GitHub). See the Nature Portfolio guidelines for submitting code & software for further information.

Data

Policy information about availability of data

All manuscripts must include a data availability statement. This statement should provide the following information, where applicable:

- Accession codes, unique identifiers, or web links for publicly available datasets
- A description of any restrictions on data availability
- For clinical datasets or third party data, please ensure that the statement adheres to our policy

Machine-readable source data for all figures, supplementary figures, and supplementary tables are provided with this paper as CSV files. The GDP, population, temperature, and tropical cyclone (TC) exposure data sets that support the findings of this study are available from https://dx.doi.org/10.5281/zenodo.8063450. National annual time series of historical per-capita GDP (GDPpc) are openly provided by the Institute for Health Metrics and Evaluation of the University of Washington (https://doi.org/10.1186/1478-7954-10-12). The national GDPpc projections are provided by the IIASA Basic Elements SSP database (https://tntcat.iiasa.ac.at/SspDb/) as part of the OECD Env-Growth model in constant 2005 US$ (PPP). The World Bank's 2024 income classification scale is openly accessible through (https://datahelpdesk.worldbank.org/knowledgebase/articles/906519).

The source of historical temperature data is the GSWP3-W5E5 historical reconstruction (https://doi.org/10.48364/ISIMIP.982724.1) which are weighted by annual, HYDEv3.3-based population densities (https://doi.org/10.48364/ISIMIP.822480.2) and using the fractional country mask (https://doi.org/10.48364/ISIMIP.635131.2) included in ISIMIP3a.

The historical TC exposure data is available from the Tropical Cyclone Exposure Database (TCE-DAT) (https://doi.org/10.5194/essd-10-185-2018) using population data from the HYDEv3.2.1 database (http://dx.doi.org/10.5194/essd-2016-58). The population projections according to the SSPs used for the projections of affected people are openly available from http://dx.doi.org/10.1088/1748-9326/11/8/084003.

The TC track simulations that were used to generate the probabilistic future TC exposure data sets are available for scientific purposes only and upon request from WindRiskTech (emai: info@windrisktech.com). The TC emulator additionally employs daily temperature data from four CMIP5 GCMs (HadGEM2-ES, MIROC5, IPSL-CM5A-LR and GFDL-ESM2M) and three RCPs (RCP4.5, RCP6.0, and RCP8.5) for the future period 2006--2105 as provided within ISIMIP2b and openly accessible through the ISIMIP data portal (https://data.isimip.org/search/tree/ISIMIP3a/tree/ISIMIP2b/InputData/climate/atmosphere/). The temperature level for the ''no further climate change`` baseline scenario is based on the HadCRUT5 global mean temperature time series that is openly accessible at the Met Office Hadley Centre (https://www.metoffice.gov.uk/hadobs/hadcrut5/data/current/download.html).
For the SCC analysis, population-weighted temperature data along the four CMIP5 GCMs and three RCPs are provided as part of the code repositories of (https://github.com/country-level-scc/cscc-paper-2018) and (https://purl.stanford.edu/wb587wt4560).

The employed country shapes are available in the public domain from the Natural Earth project web site (https://www.naturalearthdata.com/downloads/110m-cultural-vectors/110m-admin-0-countries/).
The per capita income in is openly accessible as part of the World Inequality Database (https://wid.world/bulk_download/wid_all_data.zip).

Research involving human participants, their data, or biological material

Policy information about studies with human participants or human data. See also policy information about sex, gender (identity/presentation), and sexual orientation and race, ethnicity and racism.

Reporting on sex and gender	N/A
Reporting on race, ethnicity, or other socially relevant groupings	N/A
Population characteristics	N/A
Recruitment	N/A
Ethics oversight	N/A

Note that full information on the approval of the study protocol must also be provided in the manuscript.

Field-specific reporting

Please select the one below that is the best fit for your research. If you are not sure, read the appropriate sections before making your selection.

☒ Life sciences ☐ Behavioural & social sciences ☐ Ecological, evolutionary & environmental sciences

For a reference copy of the document with all sections, see nature.com/documents/nr-reporting-summary-flat.pdf

Life sciences study design

All studies must disclose on these points even when the disclosure is negative.

Sample size	The sample size is limited by the availability of trustworthy national GDP data and satellite-based tropical cyclone observations (1981-2015). The fact that we find statistically significant results indicated that the sample size was sufficient for the analysis performed.
Data exclusions	No data were excluded
Replication	The study is a pure analysis of pre-existing data. We did not perform any kind of experiments. We provide all the data and analysis scripts needed to reproduce the regression results.
Randomization	We used boot strapping to assess uncertainties
Blinding	Does not apply, because the study objects were no individuals.

Reporting for specific materials, systems and methods

We require information from authors about some types of materials, experimental systems and methods used in many studies. Here, indicate whether each material, system or method listed is relevant to your study. If you are not sure if a list item applies to your research, read the appropriate section before selecting a response.

Materials & experimental systems

n/a	Involved in the study	
☒	☐	Antibodies
☒	☐	Eukaryotic cell lines
☒	☐	Palaeontology and archaeology
☒	☐	Animals and other organisms
☒	☐	Clinical data
☒	☐	Dual use research of concern
☒	☐	Plants

Methods

n/a	Involved in the study	
☒	☐	ChIP-seq
☒	☐	Flow cytometry
☒	☐	MRI-based neuroimaging
